# Altered Structural Covariance Among the Dorsolateral Prefrontal Cortex and Amygdala in Treatment-Naïve Patients With Major Depressive Disorder

**DOI:** 10.3389/fpsyt.2018.00323

**Published:** 2018-07-20

**Authors:** Zhiwei Zuo, Shuhua Ran, Yao Wang, Chang Li, Qi Han, Qianying Tang, Wei Qu, Haitao Li

**Affiliations:** ^1^Department of Radiology, Affiliated Southwest Hospital, Army Medical University, Chongqing, China; ^2^Department of Psychology, Affiliated Southwest Hospital, Army Medical University, Chongqing, China

**Keywords:** major depressive disorder, structural covariance, cortical thickness, subcortical volume, dorsolateral prefrontal cortex, amygdala

## Abstract

**Background:** Impairments in cognitive and emotional processing are a characteristic of major depressive disorder (MDD), and the dorsolateral prefrontal cortex (DLPFC) and amygdala are involved in these processes. However, the structural covariance between these two areas in patients with MDD has not been examined. Whether anatomical patterns are further damaged or compensated in untreated multiple-episode MDD compared to those in first-episode MDD is unclear.

**Methods:** Structural magnetic resonance imaging was performed in 35 treatment-naïve, currently depressed patients with MDD and 35 age-, sex-, and education-matched controls. The cortical thickness and subcortical volume were calculated using FreeSurfer software. Patients were divided into two subgroups based on the previous number of episodes.

**Results:** Regional abnormalities in patients with MDD were primarily observed in the frontal-limbic circuits. The negative structural association between the left DLPFC and left amygdala and the positive structural association between the bilateral DLPFC observed in controls were absent in patients with MDD. The medial orbitofrontal cortex and posterior cingulate cortex were thicker in patients with multiple-episode MDD than in patients with first-episode MDD and were positively correlated with disorder duration. No structural alterations were correlated with symptom severity.

**Conclusions:** These findings may provide structural evidence for deficits in functional networks in MDD and supports an underlying structural mechanism of dysfunction involving top-down or bottom-up processes. Morphological abnormalities in the medial orbitofrontal cortex and posterior cingulate cortex may be critical for the pathophysiological progression of multiple-episode MDD.

## Introduction

Many previous anatomical studies have focused on decreases in gray matter volume in patients with major depressive disorder (MDD). The abnormal regions reported in these studies, which are collectively known as cortical-limbic areas include the dorsolateral prefrontal cortex (DLPFC), orbitofrontal cortex (OFC), anterior cingulate cortex (ACC), posterior cingulate cortex (PCC), and amygdala (1, 2). However, these previous findings remain inconclusive (2, 3). Voxel-based morphometry, one of the most common methods used to measure volumetric changes, may actually impair the identification of subtle cortical differences because of heavy smoothing of the images and substantial cortical folding (4). Furthermore, volumetric changes are largely driven by gyrification and cortical surface area rather than cortical thickness (5), and alterations in cortical thickness are more sensitive to disease states than alterations in volume or surface area (6). In contrast to volumetric research, which consistently shows a decreasing trend in gray matter volume in patients with first-episode (FE) MDD (7), other studies have shown an increase in the thickness of several cerebral regions in untreated patients with FE MDD compared to that of controls (8–10). Based on these findings, whether a compensatory mechanism, chronic trajectory or potential age-of-onset effects participate in the pathological processes occurring during the early stage of MDD warrants further examination.

Although MDD has attracted increasing attention from the scientific community and the Chinese government, it is not recognized by most of the public. Even in a general hospital, only approximately 4% of depressed patients are identified by internists (11). Meanwhile, many depressed patients endure the disease for many years and experience multiple episodes (MEs) before seeking treatment because of the stigma and shame associated with depression in traditional Chinese culture. Without treatment, the episodes may continue and be characterized by increasingly serious symptoms. Therefore, the choice of an appropriate therapeutic schedule is more difficult for MEs compared to FEs. However, according to the result of a longitudinal observational study (12), none of the traditional demographic factors (e.g., sex, age, and socioeconomic status), clinical variables (e.g., prior episodes, age-of-onset, and episode severity) or treatment exposure (e.g., the presence or absence of treatment and treatment adequacy) are reliable predictors of recovery or recurrence in patients with MDD. Researchers have focused on examining dynamic neurobiological alterations, such as anatomical and functional deficits, some of which may be more sensitive to recurrence (13, 14). Possible structural differences between patients with FE and ME MDD, which are not clearly explored, may offer new targets for therapeutic intervention.

In the current study, we sought to use whole-brain analysis method and simultaneously attempted to make priori assumptions regarding the locations of structural deficits to systematically evaluate anatomical abnormalities. The treatment-naïve group in our study consisted of currently depressed patients with MDD to exclude the neuroprotective effects of continuous therapy and enable a direct assessment of underlying state-related changes in patients with MDD. Based on the existing literature, we hypothesized that patients with MDD would exhibit both decreased and increased cortical thickness and subcortical volumes in regions such as the DLPFC, OFC, and amygdala, and these regions may not be associated with the severity of current depressive symptoms. We also postulated that patients with ME MDD would exhibit reductions in structural measures compared with patients with FE MDD.

## Materials and methods

### Subjects

Forty-one medication-naïve, middle aged patients with MDD were recruited as potential participants from the outpatient clinic at the Department of Psychology of Southwest Hospital, Chongqing, China. All participants participated in interviews and received independent evaluations by 2 psychologists, including the 24-item Hamilton Rating Depression Scale (HAM-D_24_) (15), the Self-rating Depression Scale (SDS) (16), and the Self-rating Anxiety Scale (SAS) (17). Depression duration was assessed in an interview using the life-chart methodology. The inclusion criteria for patients were: (1) aged 18–48 years; (2) met the Diagnostic and Statistical Manual of Mental Disorders IV (DSM-IV) diagnostic criteria for MDD; (3) patients were not receiving treatment (not taking antidepressant drugs or engaged in formal psychotherapy) and currently depressed; (4) a total HAM-D_24_ score > 20 (moderate severity); (5) no history of bipolar disorder, schizophrenia, schizoaffective disorder, psychosis, bulimia, seizures, obsessive-compulsive disorder, primary post-traumatic disorder, or a current primary diagnosis of anorexia; (6) no history of alcohol abuse, substance dependence, suicidal behavior, brain injury or any contraindications for MRI; and (7) right-handedness. Thirty-five patients (22 female) met these criteria and were included in the study. Twenty of patients were currently experiencing their first depressive episode. The remaining patients had recovered from their first episode and were in the acute stage of at least their second depressive episode.

We also recruited 35 age-, sex-, and education-matched normal controls (NC) who had no history of drug dependence, psychiatric disease, traumatic brain injury, epilepsy, or chronic medical disease, such as heart failure, and no evidence to suggest an intracranial space-occupying lesion, hemorrhage, infarction, or other major neurological disease. In addition, these controls were right-handed.

All patients included in the study provided written informed consent. The study was approved by the Ethics Committee of the Southwest Hospital.

### MRI acquisition

A Siemens 3.0-Tesla Trio Tim MRI scanner (Siemens AG, Erlangen, Germany) was used to acquire structural images with a standard head coil. The subject was placed in a supine position during image acquisition. The head was fixed with sponge pads to reduce movement, and the subject was asked to keep the head as still as possible during the scan. The following magnetization-prepared rapid gradient echo acquisition parameters were used: repetition time (TR) = 1900 ms; echo time (TE) = 2.52 ms; inversion time (TI) = 1100 ms; flip angle = 9°; field of view (FOV) = 256 × 256 mm; slice thickness = 1 mm; number of slices = 176; and voxel size = 1 × 1 × 1 mm.

### MRI analysis

Structural images were subjected to volume segmentation and cortical surface reconstruction using FreeSurfer software (Massachusetts General Hospital, Boston, MA, U.S., http://surfer.nmr.mgh.harvard.edu). The post-processing procedures have been described in detail in previous studies (18, 19) and primarily consisted of the following steps: Talairach coordinate system conversion, bias-field correction, signal strength standardization, removal of the skull and soft tissues, automated volume partitioning and white matter segmentation, topology correction, and determination of the gray-white matter and leptomeningeal tissue boundaries. Inflated brain surfaces and cortical thicknesses were obtained. These post-processing procedures were performed separately on each cerebral hemisphere. Cortical thickness was defined as the shortest straight-line distance between the pial surface and the gray-white matter boundary. The volumes of subcortical regions, including the thalamus, caudate, putamen, pallidum, and amygdala, were extracted. As researchers are still debating whether hippocampal volumes are reduced in patients with MDD (20–22), we further segmented the hippocampus to determine whether structural variations in hippocampal subfields play a role in patients with MDD. The segmentations of the hippocampus include the fimbria, presubiculum, subiculum, cornu ammonis (CA) 1, CA2/3, CA4/dentate gyrus (DG) fields and the hippocampal fissure (23).

### Statistical analyses

First, we compared the demographic and clinical features and hemispheric cortex measurements between patients with MDD and NCs. The Mann-Whitney *U*-test or independent sample *t*-test were used for parameters that were not normally distributed (i.e., age, education level) and parameters with a normal distribution, (e.g., HAM-D_24_ score, SDS score, and SAS score), respectively. The chi-square test was used to assess differences in sex distribution. Differences in cortical thickness between the patients with MDD and NCs were then evaluated using the vertex-wise general linear model and a whole-brain statistical threshold correction was performed using the Monte Carlo simulation method. Statistical significance was set at a cluster-wise corrected *P*-value < 0.05. The average cortical thickness of the significant clusters was calculated for every subject to obtain a regionally specific comparator. Specifically, eight regions of interest (ROIs), including the bilateral DLPFC, OFC, ACC, and PCC, were created based on the Desikan template (24) and previous research (25) to extract the average cortical thickness. Between-group differences in average cortical thickness of the ROIs were assessed using an independent samples *t*-test, and differences in subcortical volumes were assessed using the Mann-Whitney *U*-test. Next, the MDD group was divided into two subgroups according to the number of episodes, i.e., the FE group and the ME group. The differences in average cortical thickness and subcortical volume in all regions observed between the MDD and NC groups in the previous statistical comparison were examined in the FE and ME groups using the independent sample *t*-test. Finally, correlation analyses were performed to explore the relationships among brain structures and clinical features. Structural covariance was examined with Pearson correlation analysis to determine the structural relationships between the left DLPFC and left amygdala, right DLPFC and right amygdala, left DLPFC and right DLPFC, and left amygdala and right amygdala. Then, Snedecor's method (26) was used to transform *r*-values to *z*-values to evaluate the significant differences in correlation coefficients between patients with MDD and NCs. False discovery rate (FDR) correction was applied to between-group analyses and correlation analyses that involved multiple comparisons.

## Results

### Participants' characteristics and hemispheric measures

Table 1 shows the demographic information, clinical data and hemispheric measurements for the MDD and NC groups. The two groups were matched in terms of age, sex, and education (*P* > 0.05). As expected, the patients with MDD had higher HAM-D_24_ scores, SDS scores, and SAS scores than the NCs. The patients with MDD showed a nearly significant trend for the increase in the average cortical thickness of the left hemisphere (*t* = 1.987, *P* = 0.052). No significant differences in the average cortical thickness of the right hemisphere and total subcortical volume were observed between the MDD and NC groups.

### Surface-based cortical thickness analysis

**Figure 1:**
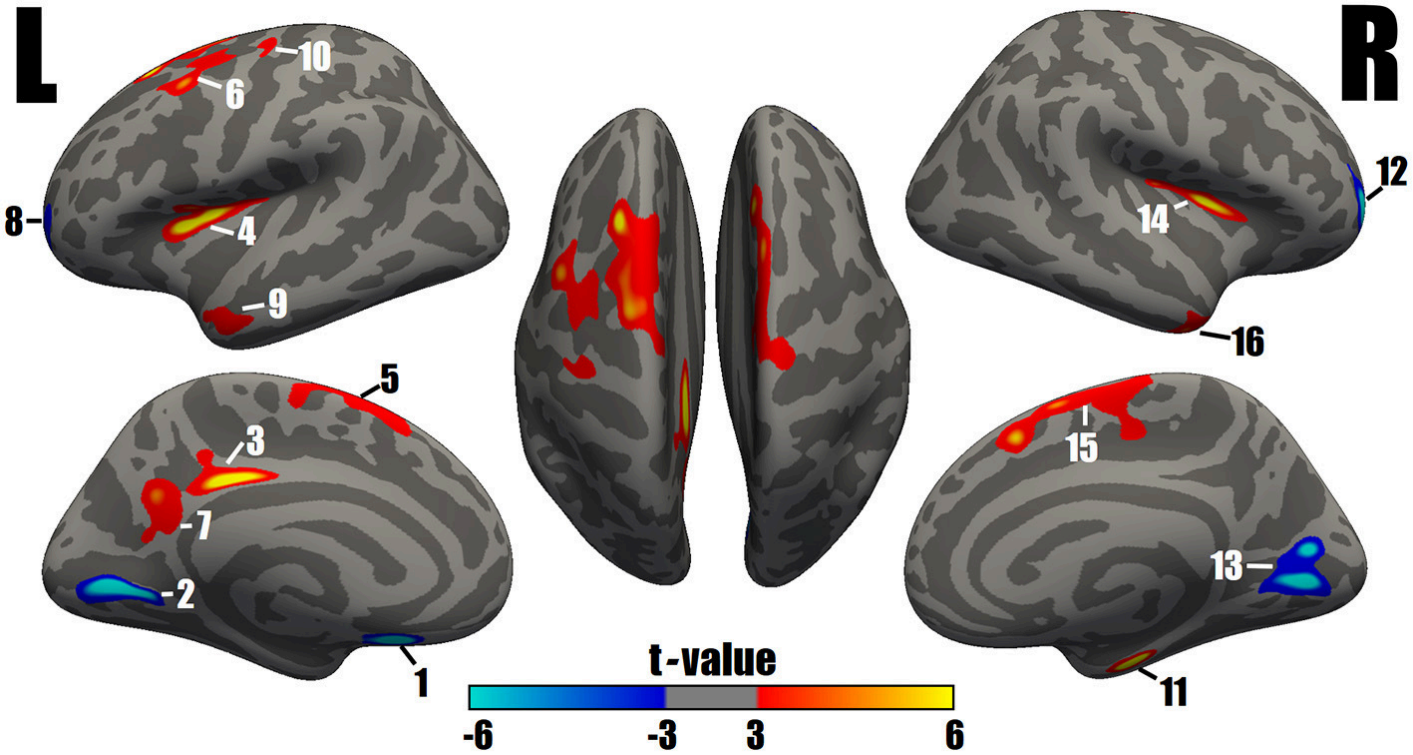
Surface maps of significant differences in cortical thickness between patients with MDD and NCs. As determined by the GLM analysis, differences are presented on an inflated cortical surface (corrected *P*-value < 0.05). Dark gray indicates a gyrus; light gray indicates a sulcus. “L” indicates the lateral, medial and dorsal surfaces of the left hemisphere. “R” indicates the lateral, medial and dorsal surfaces of the right hemisphere. The color bar represents the *t* values from −6 to −3 and 3 to 6. Blue indicates cortical thinning in the MDD group compared with the NC group; a closer proximity to sky blue indicates a greater difference. Red to yellow indicates cortical thickening in the MDD group compared with the NC group; a closer proximity to yellow indicates a greater difference. The numerals refer to the cluster numbers listed in Table 2.

A comparison of the cortical thickness between the MDD and NC groups showed relatively symmetrical changes in 16 clusters (Figure 1 and Table 2), with both significant increases and decreases in cortical thickness observed. The largest and most significant increases in thickness were observed in the bilateral insula, superior frontal cortex, middle temporal gyrus, left PCC, caudal middle frontal cortex, precuneus, precentral gyrus, and right entorhinal cortex. The regions with significantly decreased thickness were the bilateral rostral middle frontal cortex, left lingual gyrus, medial orbitofrontal cortex (MOFC), and right pericalcarine cortex (Figure 1 and Table 2).

### ROI-based cortical thickness analysis

**Figure 2:**
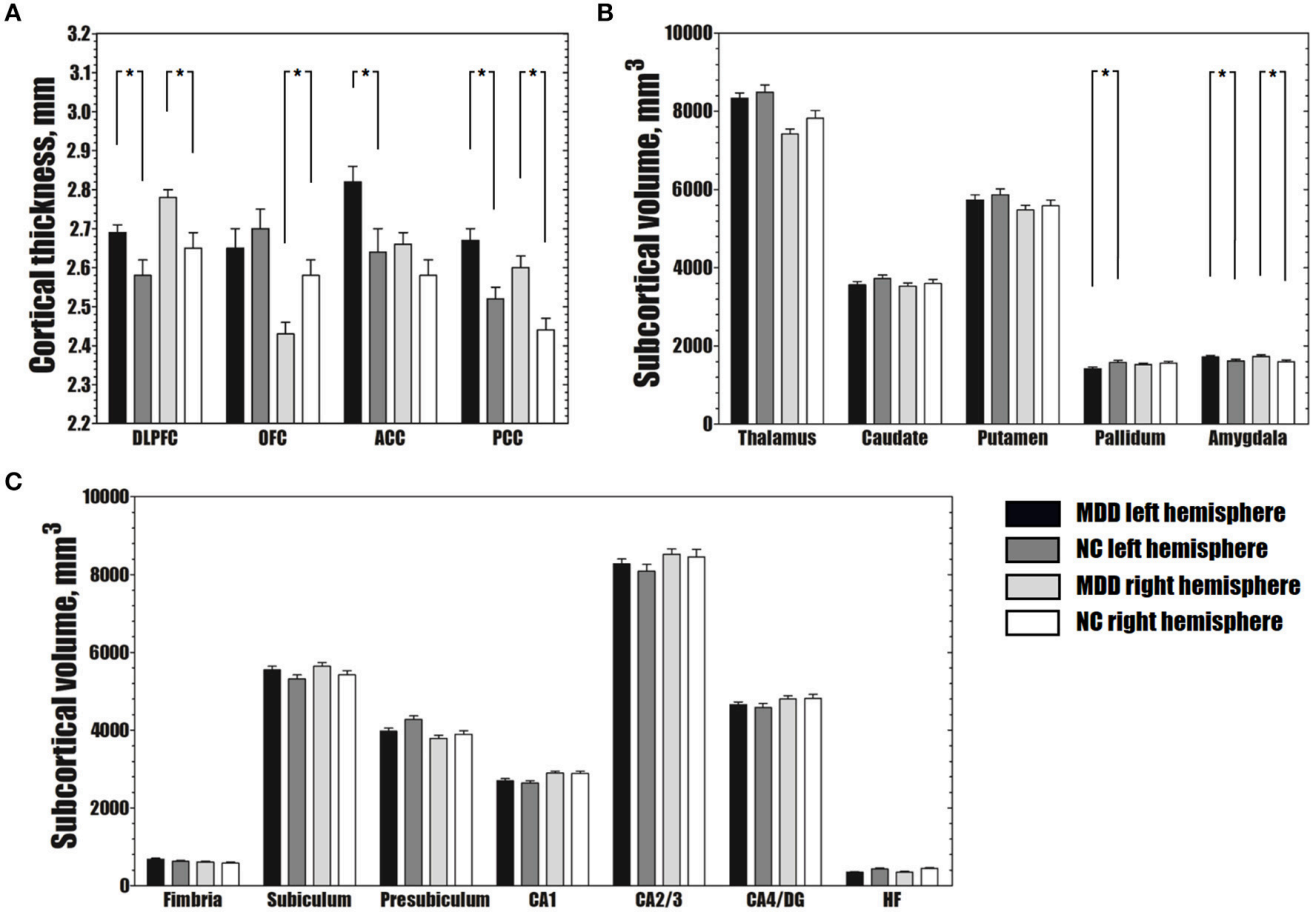
Differences in average cortical thickness **(A)**, subcortical volume **(B)** and volume of hippocampal subfields **(C)** between the MDD and NC groups. **(A)** The MDD group shows greater average cortical thickness in the bilateral DLPFC, left ACC and bilateral PCC, and smaller average cortical thickness in the right OFC than the NC group (FDR-corrected *P* < 0.05). **(B)** The MDD group has a larger bilateral amygdala and smaller left pallidum than the NC group (FDR-corrected *P* < 0.05). **(C)** No significant differences were observed in the volumes of hippocampal subfields between the MDD and NC groups (FDR-corrected *P* > 0.05). The error bars indicate standard errors. ^*^FDR-corrected *P* < 0.05. MDD, major depressive disorder; NC, normal controls; DLPFC, dorsolateral prefrontal cortex; OFC, orbitofrontal cortex; ACC, anterior cingulate cortex; PCC, posterior cingulate cortex; CA, cornu ammonis; DG, dentate gyrus; HF, hippocampal fissure; FDR, false discovery rate.

Abnormal structural changes in the DLPFC, OFC, ACC, and PCC in patients with MDD have been reported in many structural studies (1, 2). Therefore, we further calculated the average cortical thickness of these cortical areas using the ROI method. The bilateral DLPFC, left ACC, and bilateral PCC were thicker in patients with MDD than in NCs (FDR-corrected *P* < 0.05), and the right OFC was thinner in patients with MDD than in NCs (FDR-corrected *P* < 0.05) (Figure 2A).

### Subcortical volume analysis

Compared to the NC group, the MDD group showed lower volumes in the left pallidum (FDR-corrected *P* < 0.05). Greater subcortical volumes were detected in the bilateral amygdala (FDR-corrected *P* < 0.05) (Figure 2B).

In the hippocampal subfields analysis, no significant differences in the volumes of any of the seven hippocampal subfields were found between the MDD and NC groups (FDR-corrected *P* > 0.05) (Figure 2C).

### Subgroup analysis

As shown in Table 1, no differences in sex, age, age-of-onset, education, HAM-D_24_ score, SAS score, SDS score, hemispheric cortical thickness or subcortical volume were observed between the subgroups (FE vs. ME). No significant differences in subcortical volumes were observed in the subgroup comparison of FE and ME MDD patients. Surprisingly, cluster 1 and cluster 3 were thicker in the ME group than in the FE group (FDR-corrected *P* < 0.05). The peak vertexes of clusters 1 and 3 are located in the MOFC and PCC, respectively.

**Table 1:** Demographic features and hemispheric cortex measures.

**Characteristic**	**MDD (*n* = 35)**	**NC (*n* = 35)**	**Diagnosis effect**	***P* value**	**FE (*n* = 20)**	**ME (*n* = 15)**	**Diagnosis effect**	***P***
**DEMOGRAPHIC/CLINICAL CHARACTERISTICS**								
Age, years	28.91 ± 1.57	28.11 ± 1.15	*u* = −0.218	0.827	27.40 ± 1.90	30.93 ± 2.64	*t* = −1.115	0.273
Age-of-onset, years	27.00 ± 1.53	–	–	–	26.45 ± 1.92	27.73 ± 2.56	*t* = −0.410	0.684
Sex, female: male	22:13	20:15	χ^2^ = 0.238	0.626	13:7	9:6	χ^2^ = 0.092	0.762
Education, years	13.71 ± 0.51	13.91 ± 0.61	*u* = −0.336	0.737	13.80 ± 0.66	13.60 ± 0.83	*t* = 0.191	0.850
Duration of disorder, years	1.65 ± 0.26	–	–	–	0.57 ± 0.07	3.10 ± 0.32	*t* = −7.737	< 0.0001
HAM-D_24_ score	30.45 ± 0.68	2.54 ± 0.26	*t* = 38.142	< 0.0001	28.80 ± 0.97	30.93 ± 1.40	*t* = −1.295	0.204
SAS score	56.49 ± 1.94	27.46 ± 0.27	*t* = 14.846	< 0.0001	58.15 ± 2.46	54.20 ± 3.08	*t* = 1.014	0.318
SDS score	61.77 ± 1.57	27.51 ± 0.26	*t* = 21.465	< 0.0001	63.30 ± 1.87	60.80 ± 2.42	*t* = 0.833	0.411
**HEMISPHERIC CORTEX MEASURES**								
Mean cortical thickness of LH, mm	2.56 ± 0.01	2.51 ± 0.02	*t* = 1.987	0.052	2.54 ± 0.02	2.58 ± 0.02	*t* = −1.671	0.104
Mean cortical thickness of RH, mm	2.54 ± 0.01	2.50 ± 0.02	*t* = 1.380	0.174	2.53 ± 0.02	2.56 ± 0.02	*t* = −1.107	0.276
Total subcortical volume of LH, mm^3^	25789 ± 368	26330 ± 426	*t* = −0.964	0.339	25628 ± 347	26003 ± 737	*t* = −0.460	0.650
Total subcortical volume of RH, mm^3^	24883 ± 387	25244 ± 482	*t* = −0.584	0.561	24719 ± 373	25103 ± 768	*t* = −0.451	0.657

*MDD, major depressive disorder; NC, normal control; FE, first episode; ME, multiple episode; HAM-D_24_, 24-item Hamilton Depression Scale; SAS, Self-rating Anxiety Scale; SDS, Self-rating Depression Scale; LH, left hemisphere; RH, right hemisphere*.

*Continuous variables are expressed as mean ± SEM*.

*Significance P-value < 0.05*.

*To test differences in age, and education between MDD patients and NCs, Mann-Whitney U-test was applied*.

*To test differences in HAM-D_24_ score, SAS score, SDS score, mean cortical thickness, and total subcortical volume between MDD patients and NCs, independent sample t-test was applied*.

*To test differences in age, age of onset, education, duration of disorder, HAM-D24 score, SAS score, SDS score, mean cortical thickness, and total subcortical volume between FE and ME groups, independent sample t-test was applied*.

*To test the distribution of female/male chi-square test was computed*.

**Table 2:** Surface-based cluster summary of significant cortical changes in patients with MDD.

**Cluster Number**	***t*-value Max**	**Size (mm^2^)**	**MNI coordinates of peak vertex**			**CWP**	**CWPLow**	**CWPHi**	**Anatomical location**
			***X***	***Y***	***Z***				
**LH**									
1	−7.834	222.51	−5.9	21.3	−20.8	0.0193	0.0169	0.0219	Medial orbitofrontal cortex
2	−7.395	711.89	−6.5	−74.1	4.3	0.0002	< 0.0001	0.0004	Lingual gyrus
3	7.150	369.94	−5.0	−32.6	33.2	0.0004	< 0.0001	0.0008	Posterior cingulate cortex
4	7.001	553.11	−30.7	9.5	9.4	0.0002	< 0.0001	0.0004	Insula
5	6.577	1409.82	−23.0	23.3	54.5	0.0002	< 0.0001	0.0004	Superior frontal cortex
6	5.481	652.21	−39.2	7.7	52.9	0.0002	< 0.0001	0.0004	Caudal middle frontal cortex
7	5.283	435.36	−8.7	−58.6	26.8	0.0002	< 0.0001	0.0004	Precuneus
8	−5.114	304.83	−22.1	52.0	−3.8	0.0030	0.0020	0.0040	Rostral middle frontal cortex
9	4.600	400.80	−55.0	−0.0	−29.4	0.0002	< 0.0001	0.0004	Middle temporal cortex
10	3.930	211.57	−34.8	−20.0	64.9	0.0232	0.0205	0.0260	Precentral gyrus
**RH**									
11	8.020	233.78	22.5	−12.4	−30.5	0.0140	0.0118	0.0161	Entorhinal cortex
12	−7.518	680.38	23.2	51.3	−0.6	0.0002	< 0.0001	0.0004	Rostral middle frontal cortex
13	−7.217	1061.45	8.1	−74.3	5.0	0.0002	< 0.0001	0.0004	Pericalcarine cortex
14	6.449	312.31	33.2	2.8	11.6	0.0018	0.0010	0.0026	Insula
15	5.843	1098.42	8.2	17.9	49.1	0.0002	< 0.0001	0.0004	Superior frontal cortex
16	5.058	334.41	44.7	−0.5	−33.0	0.0016	0.0010	0.0024	Middle temporal cortex

*CWP, cluster wise P-value; MNI, Montreal Neurological Institute; LH, left hemisphere; RH, right hemisphere*.

*CWPLow and CWPHi: 90% confidence interval for CWP*.

### Correlation analysis

Among the clinical characteristics, only a significant positive correlation between the SAS score and SDS score (*r* = 0.589, *P* < < 0.0001) was detected.

In patients with MDD, no changes in cortical thickness, or subcortical volume in clusters or areas were significantly correlated with symptom severity (using the HAM-D_24_, SDS, and SAS scores). However, the average cortical thickness of clusters 1 and 3 showed positive correlations with disease duration in the MDD group (FDR-corrected *P* < 0.05).

Structural covariance analyses were specifically performed among the DLPFC and amygdala in the MDD and NC groups. In NCs, the left DLPFC—left amygdala and the right DLPFC—right amygdala showed significantly negative correlations; the left DLPFC—right DLPFC and the left amygdala—right amygdala showed significantly positive correlations. In the patients with MDD, only the left amygdala—right amygdala showed a significantly positive association (Table 3). Using Snedecor's method (26), we further found differences in the correlation coefficients (*r***-**values) of the left DLPFC—left amygdala and the left DLPFC—right DLPFC between the MDD and NC groups (Table 3).

**Table 3:** Comparison results of correlation coefficients of the DLPFC-amygdala between MDD and NC groups.

	**Correlation analysis**					
	**MDD**		**NC**		***Z***	**FDR-corrected *P***
	***r***	**FDR-corrected *P***	***r***	**FDR-corrected *P***		
L DLPFC - L amygdala	0.084	0.632	−0.466	0.007	2.357	0.037
R DLPFC - R amygdala	−0.326	0.112	−0.346	0.042	0.09	0.928
L DLPFC - R DLPFC	0.281	0.136	0.884	< 0.0001	−4.42	< 0.0001
L amygdala - R amygdala	0.760	< 0.0001	0.808	< 0.0001	−0.5	0.823

*MDD, major depressive disorder; NC, normal control; FDR, false discovery rate; L, left; R, right; DLPFC, dorsolateral prefrontal cortex*.

## Discussion

Consistent with our hypotheses, prominent thickening and thinning was observed in specific cortical regions in patients with MDD. Although significant differences in cortical thickness at the hemispheric level were not observed, patients with MDD had a nearly significant trend toward an increase in the average cortical thickness of the left hemisphere. Thus, cortical thickening and not thinning might distinguish the groups. Subcortical regions also showed volumetric abnormalities in both directions in the MDD group; however, the total subcortical volume did not display any significantly changes. Therefore, the volumetric changes in subcortical regions at the hemispheric level were relatively balanced.

Based on the results of our study, altered cortical thickness and subcortical volumes of brain regions, such as the precentral gyrus, rostral middle frontal cortex, superior frontal cortex, MOFC, insula, amygdala, ACC, entorhinal cortex, and pallidum, are involved the five frontal-basal ganglia circuits (27, 28) (the motor circuit, oculomotor circuit, dorsolateral prefrontal circuit, orbitofrontal circuit and anterior cingulate circuit). These frontal-basal ganglia circuits play important roles in motor activity and human behavior. The dorsolateral prefrontal circuit mainly mediates executive function, and dysfunction of this circuit may produce impairments in retrieving remote memories, managing actions according to an external stimulus, altering behaviors appropriately, and mental flexibility. Subjects with dysfunction of the orbitofrontal circuit and anterior cingulate circuit exhibit personality changes including behavioral disinhibition, emotional lability, and reduced motivation (28). These regionally specific characteristics of structural changes in our study convincingly supported that MDD could involve deficits in neural networks across the whole brain. Grieve et al. (2) also found widespread gray matter volume reductions in a large sample of MDD patients. However, some participants in their study may have had a history of antidepressants uses, which could have an important impact on brain structures. It should be noted that cortical volume is composed of cortical surface area and cortical thickness, and decreased cortical volume could be coupled with increased cortical thickness.

Unlike the distributed cluster results, even when no statistical differences in regional areas were present between the two groups, the average cortical thickness could be significantly changed due to the accumulation of minor alterations. Therefore, comparison of the average cortical thickness of a brain subarea can reflect integral alterations in this area. Thus, we measured the average cortical thickness of the DLPFC, OFC, ACC, and PCC, which play key roles in the frontal-basal ganglia circuits. Our results demonstrated that the right OFC thickness and the left pallidum volume were decreased, while the thickness of the bilateral DLPFC, left ACC and bilateral PCC and the volume of bilateral amygdala were increased in patients with MDD. The OFC connects the frontal monitoring systems (e.g., the DLPFC) to the limbic system (e.g., cingulate, amygdala) (29), and the pallidum is involved in the five parallel frontal-basal ganglia circuits (28). Therefore, one possible explanation for these alterations could be dysfunction of the OFC and pallidum, which might lead to abnormalities in connectivity among cortical-limbic areas. Without sufficient inhibitory control, the amygdala activity in depressed patients is usually increased in response to emotional stimuli (30). To compensate for this less efficient self-regulation, the DLPFC and cingulate are recruited to a greater degree. Such a compensatory mechanism was found in remitted MDD patients who had a thicker PCC than non-remitting patients (4).

The altered relationship among the DLPFC and amygdala was an another critical factor underlying the impairment in functional connectivity in patients with MDD. Although the thickness of the bilateral DLPFC and the volume of the left amygdala were greater, the negative relationship between the left DLPFC and left amygdala and the positive relationship between the left DLPFC and right DLPFC were absent, suggesting that the inverse reciprocity between the ipsilateral DLPFC and amygdala and the synergistic pattern between the bilateral DLPFC in patients with MDD were impaired. Cognitive-control and emotional-processing circuitry usually work in opposition to each other, and disharmony between the two areas may also be present in normal individuals, such as adolescents. Because different cerebral regions follow unique maturational trajectories during brain growth and development, with cortical maturity lagging behind that of subcortical region (31), healthy adolescents often exhibit impetuous but deficient top-down (cortical-to-limbic) cognitive control or intensive bottom-up (limbic-to-cortical) emotional processing (32). However, resting-state fMRI studies have found that adolescents with MDD may show decreased bottom-up connectivity or an increased imbalance in resting-state functional activity in frontal-subcortical circuits (32, 33). In a study on adult subjects (34), patients with depression showed enhanced amygdala responses and failure to recruit the DLPFC when exposed to affective distracters during cognitive tasks. As shown in another study (35), depressed individuals showed a positive association between the prefrontal cortex and amygdala during an affective task, and an opposite association was observed in controls. However, antidepressant treatment can reverse the functional patterns and connectivity impairments of depression by decreasing limbic activity in response to a negative stimulus and increasing cortical-limbic connectivity (36, 37). Similarly, chronic therapies with different antidepressants can block or reverse neuronal atrophy and cell loss in several cerebral regions such as prefrontal cortex and amygdala through increasing expression of neurotrophic factors (38). In a longitudinal study (39), remitted patients who received intensive antidepressant therapy showed a pattern of increasing cortical thickness in the OFC, DLPFC and inferior temporal gyrus over follow-up. These findings indicated that dysregulation of bottom-up emotional processing and top-down cognitive control are crucial features underlying the pathophysiology of MDD and that these features can explain why depressed individuals tend toward the processing of negative emotion such as fear, sadness or anxiety (40). Therefore, our work provides further evidence for a potential morphological basis of disorganized regional interactions in cortical-limbic circuits in patients with MDD.

Few structural MRI studies have analyzed hippocampal subregions in untreated patients with MDD. A meta-analysis (41) confirmed that only patients with MDD who with a disease duration longer than 2 years or more than 1 disease episode displayed smaller hippocampal volume than controls. The mean duration of MDD in patients in our study was approximately 1.65 years, which could explain why we could not find significant differences in hippocampal subfield volumes. Travis et al. (42) and Na et al. (43) also did not find differences in hippocampal subfield volumes between MDD patients and controls. However, they found correlations between volumes of specific hippocampal subregions and glucocorticoid receptor methylation and cortisol levels were altered in patients with MDD. Therefore, the structural pathophysiological process of hippocampal subregions in MDD patients whose disorder duration is less than 2 years or that in FE patients need to be further explored.

Another important goal of this study was to assess morphometric changes in patients with MDD who experienced multiple depressive episodes but were never treated. Although the main cortical change in patients with MDD has been consistently shown to be a reduction in the thickness (21, 8, 44) or volume (45, 2, 22, 3, 7), an increasing number of studies have recently reported increases in cortical thickness in untreated patients with FE MDD (9, 46, 10). Our results further verify that cortical thickness is also increased in untreated patients with ME MDD. The increased cortical thickness may reflect the early course of a chronic pathological trajectory that will eventually result in reduction of cortical volume (8). However, it may be a result of the compensatory response of plastic neurons, glia or neuropils. Considering that no difference in age was found between the FE and ME groups in our study, theoretically, the FE patients would have a later age-of-onset than ME patients, which might impact cortical thickness. However, the two groups did not show a significant difference in age-of-onset, possibly due to the relatively small sample size. Regardless of the mechanism, these changes may be related to inflammation, the hypothalamic-pituitary (HPA)-axis, or neurogenesis (47, 48, 9). Additionally, the severity of depression was not greater in the participants with ME MDD than in patients with FE MDD, and MEs may lead to thickening of the MOFC and PCC. Moreover, cortical thickness and subcortical volume did not exhibit significant changes as depressive symptoms increased in the patients with MDD, which is consistent with previous studies (49, 2, 46). Nevertheless, the left MOFC and PCC displayed positive relationships with the disease duration. Therefore, dynamic cerebral morphometry may be a more reliable and continuous measure of disease progression in patients with MDD than traditional demographic and clinical variables.

Increases in the thickness of the MOFC and PCC in patients with ME MDD may reveal pivotal pathophysiological mechanisms of the ME process. Patients with MDD often show an increase in self-focused behaviors. The self-reflective function of the MOFC and PCC was proven to have two dissociated components using functional techniques. Specifically, the MOFC is related to a more inward-directed focus (e.g., hope and aspirations), whereas the PCC is related to a more outward-directed, social focus (e.g., duties and obligations) (50). In task-negative networks, Zhou et al. (51) observed increased functional connectivity of the MOFC and PCC in patients with MDD, which may reflect a potential basis for the negative bias in emotional processing. According to the results of a resting-state fMRI study, functional dysconnectivity is linked to local cortical thinning in patients with MDD (44). Therefore, we speculate that reinforced connectivity may be associated with increased thickness of the MOFC and PCC, but this hypothesis requires further verification.

Several limitations of this study should be noted. First, our sample size was relatively small, so any morphometric abnormalities that we identified must be interpreted with caution. A larger number of patients might be useful in obtaining more robust results. Second, this study was a cross-sectional investigation, and potential variations in the duration of illness should be studied longitudinally in future studies. Third, the actual number of depressive episodes was not recorded in the present study; thus, we were unable to assess any relationships between structural changes and the number of depressive episodes. Furthermore, although none of the depressed participants had received treatment, some results may be exaggerated or hidden because patients with FE and ME MDD were combined for certain analyses. Further studies that combine other neuroimaging methods, such as resting-state fMRI and DTI, are needed to explore the associations between the functional and structural changes that underlie impairments in top-down and bottom-up regulation in patients with MDD.

In conclusion, the present study complements and extends previous anatomical studies of patients with MDD through a surface-based approach and shows that structural alterations in untreated patients with MDD are primarily located in the frontal-basal ganglia circuits, which may provide a structural evidence for deficits in functional networks involved in MDD. The finding of a lack of correlation within DLPFC and amygdala in patients with MDD supports an underlying structural mechanism for dysregulation of top-down or bottom-up processes. Moreover, dynamic changes in morphology were observed during the progression of MDD, which may be a more reliable measure than traditional clinical variables, and alterations in the MOFC and PCC may represent a critical pathophysiological mechanism in the progression of ME MDD. These findings contribute to improving our understanding of the neurobiology and pathophysiology of MDD and offer potential targets for the development of more effective treatments for this condition.

## Author contributions

HL and WQ designed experiments. SR, QT, and ZZ carried out experiments. ZZ, SR, YW, CL, and QH analyzed imaging results. ZZ and HL wrote the manuscript. All authors contributed to manuscript revision, read and approved the submitted version.

### Conflict of interest statement

The authors declare that the research was conducted in the absence of any commercial or financial relationships that could be construed as a potential conflict of interest.

## Abbreviations

MDD: major depressive disorder
DLPFC: dorsolateral prefrontal cortex
OFC: orbitofrontal cortex
ACC: anterior cingulate cortex
PCC: posterior cingulate cortex
CA: cornu ammonis
DG: dentate gyrus
FDR: false discovery rate.
